# Non-Invasive Assessment of Mild Stress-Induced Hyperthermia by Infrared Thermography in Laboratory Mice

**DOI:** 10.3390/ani12020177

**Published:** 2022-01-12

**Authors:** Urša Blenkuš, Ana Filipa Gerós, Cristiana Carpinteiro, Paulo de Castro Aguiar, I. Anna S. Olsson, Nuno Henrique Franco

**Affiliations:** 1 Royal (Dick) School of Veterinary Studies, Easter Bush Campus, The University of Edinburgh, Edinburgh EH25 9RG, UK; ursa.blenkus@i3s.up.pt; 2 Laboratory Animal Science, i3S—Instituto de Investigação e Inovação em Saúde, Universidade do Porto, Rua Alfredo Allen 208, 4200-135 Porto, Portugal; olsson@ibmc.up.pt; 3 Neuroengineering and Computational Neuroscience Group, i3S—Instituto de Investigação e Inovação em Saúde, Universidade do Porto, Rua Alfredo Allen 208, 4200-135 Porto, Portugal; anafgeros@ineb.up.pt (A.F.G.); crscarpinteiro@gmail.com (C.C.); pauloaguiar@ineb.up.pt (P.d.C.A.); 4 FEUP—Faculdade de Engenharia da Universidade do Porto, 4200-465 Porto, Portugal

**Keywords:** stress, anxiety, stress-induced hyperthermia, infrared thermography, laboratory mice, mouse handling

## Abstract

**Simple Summary:**

Stressful events can trigger body temperature variations in mammals. The most commonly used methods for measuring temperature in laboratory mice are stressful and invasive in nature, and can themselves cause stress-induced hyperthermia (SIH). This raises concerns regarding both animal welfare and research output. Infrared thermography (IRT) offers a non-invasive alternative, if proven to accurately identify SIH. We exposed mice to mild handling-induced stress, by either tail-picking or the reportedly less-impactful tunnel-handling technique. Temperature was measured by reading microchip devices (PIT-tags) implanted subcutaneously (T_sc_), and by a thermal camera to measure mean body surface temperature (T_body_) and mean tail surface temperature (T_tail_). As expected, during acute stress exposure, both T_sc_ and T_body_ increased, while T_tail_ decreased. No differences in stress-induced hyperthermia were found between the two handling techniques. This suggests that such differences may not be detectable in the context of co-occurring stressful events, such as opening of the cage lid, exposure to light, or presence of the handler. Within the same cage, animals handled last consistently showed higher body temperatures than those handled first, raising the issue of minding the order by which animals are tested. Our results suggest IRT offers a reliable non-invasive method for assessing SIH in laboratory rodents.

**Abstract:**

Stress-induced hyperthermia (SIH) is a physiological response to acute stressors in mammals, shown as an increase in core body temperature, with redirection of blood flow from the periphery to vital organs. Typical temperature assessment methods for rodents are invasive and can themselves elicit SIH, affecting the readout. Infrared thermography (IRT) is a promising non-invasive alternative, if shown to accurately identify and quantify SIH. We used in-house developed software ThermoLabAnimal 2.0 to automatically detect and segment different body regions, to assess mean body (T_body_) and mean tail (T_tail_) surface temperatures by IRT, along with temperature (T_sc_) assessed by reading of subcutaneously implanted PIT-tags, during handling-induced stress of pair-housed C57BL/6J and BALB/cByJ mice of both sexes (N = 68). SIH was assessed during 10 days of daily handling (DH) performed twice per day, weekly voluntary interaction tests (VIT) and an elevated plus maze (EPM) at the end. To assess the discrimination value of IRT, we compared SIH between tail-picked and tunnel-handled animals, and between mice receiving an anxiolytic drug or vehicle prior to the EPM. During a 30 to 60 second stress exposure, T_sc_ and T_body_ increased significantly (*p* < 0.001), while T_tail_ (*p* < 0.01) decreased. We did not find handling-related differences. Within each cage, mice tested last consistently showed significantly higher (*p* < 0.001) T_sc_ and T_body_ and lower (*p* < 0.001) T_tail_ than mice tested first, possibly due to higher anticipatory stress in the latter. Diazepam-treated mice showed lower T_body_ and T_sc_, consistent with reduced anxiety. In conclusion, our results suggest that IRT can identify and quantify stress in mice, either as a stand-alone parameter or complementary to other methods.

## 1. Introduction

Body temperature variation is potentially useful as an indicator of stress in laboratory animals, as stressful situations elicit a quick onset of body temperature increase in several animal species, including mice [1]. Responses to acute stressors include activation of the sympathetic-adreno-medullar (SAM) axis, providing a rapid physiological response, such as the secretion of noradrenaline and norepinephrine, and in the second stage, activation of the hypothalamus-pituitary-adrenal (HPA) axis, promoting the secretion of glucocorticoids [2]. These responses amongst others additionally include rapid cardiovascular system activation [3] and heat production [4].

Body temperature in mice is traditionally measured with the use of thermometers, either rectal or infrared, or by radiotelemetry devices. Using rectal thermometers requires the removal of each mouse from the home cage, handling, movement restraint and rectal probe insertion. Infrared thermometers are less invasive, but also require restraining, and are typically less likely to yield accurate and consistent readings [5]. As repeated handling and alarm calls elicited by handled animals to cage mates is shown to cause SIH [6,7,8], this method can therefore impact the accuracy of the results [6]. While radiotelemetry devices can be measured remotely, they have to be surgically implanted, which can cause inflammation [9], lesions, long-lasting behavioural and physiological changes [10] and up to 10% mortality [11]. Passive integrated transponder (PIT) tags are smaller and less invasive than battery-operated sensors, but require reading at close range, often also requiring picking up animals, thus defeating the purpose of contactless measurement. Transponders can also move from the desired position [7], stop functioning [12] or be removed by the animal [5]. The invasiveness of the above-mentioned methods can therefore alter the experimental outcome, while also impacting the welfare of the animals.

Infrared thermography (IRT) is a potential non-invasive alternative for identifying and quantifying SIH, by estimating variations in animal body surface temperature. With increasing affordability of this technology, its use in laboratory animal research has also been increasing, including for following infection progress [13], identifying housing problems [14], monitoring cold stress [15], identifying coping styles [16], or monitoring fear [17,18] and anxiety responses [19].

SIH in laboratory rodents causes an increase in body and eye temperature, while temperature in the extremities, such as in the tail and paws, tends to decrease [18,20]. These contrasting responses during acute stress exposure are mostly mediated by either vasodilation or vasoconstriction regulating blood flow into the different body regions, each representing a ‘thermal window’ that offers specific information for the understanding of the general physiological response [21,22,23]. For laboratory animals, the tail, ocular, auricular and interscapular regions are the most commonly observed thermal windows [22]. Eye temperature has been proposed as the closest approximation to core temperature [24]. However, clear images of the eye are hard to obtain, resulting in up to 40% of missing data [25] or the need to restrain the animals [24]. Therefore, full body surface temperature (more specifically, of the dorsal surface) has been proposed as a more robust measurement for following temperature variations in free-moving mice [25]. The dorsal side of the tail can also be easily observed, yet there is low inter-observer agreement on what defines the ‘base of the tail’ leading to variability during assessment by IRT [25]. Moving to an objective method, such as automated assessment of mean tail temperature by IRT, would remove this observer bias. Given the thermoregulatory importance of the tail—which presents ideal anatomical features for this role, namely its large surface/volume ratio, its furlessness, and its particular vascularization [26]—IRT measurements of the tail temperature would provide more precise data on mouse thermal physiology during stress.

The aim of this study was to assess the ability of IRT to detect handling induced SIH in mice, and compare SIH in response to different handling techniques, both during daily handling and behavioural tests of anxiety. Tunnel-handling of mice has been shown to elicit lower levels of anxiety during behavioural tests than tail-picking [27], and this effect is already evident after exposure to short handling periods during routine cage changes or brief (2 s) daily handling for the duration of 10 days, without prior habituation to handling techniques [28]. We hypothesized that: (I) We would be able to detect hyperthermic stress response in mice using IRT. (II) We would be able to detect lower SIH in mice picked up by means of a tunnel, as compared to mice picked up by the tail, (III) which would be consistent for different sexes and strains. (IV) Temperature measured as mean body surface temperature (T_body_) and mean tail surface temperature (T_tail_) by IRT would be comparable with temperature measured by reading of subcutaneously implanted thermosensitive PIT-tags (T_sc_), a non-contact approach to avoid the impact of temperature assessment methods themselves. (V) Temperature variations that indicate stress intensity would be in agreement with results from the elevated plus maze (EPM) and voluntary interaction test (VIT). Should thermal responses result from anxiety, rather than increased motor activity, (VI) we also hypothesized that SIH would be less pronounced in animals receiving an acute dose of an anxiolytic drug (diazepam, shown to reduce anxiety levels in mice during behavioural tests [1,29]), prior to EPM, for both tunnel-handled, and tail picked mice.

## 2. Materials and Methods

### 2.1. Animals and Housing Conditions

We used 68 mice of two strains (BALB/cByJ and C57BL/6J) and both sexes, bred in-house under specific-pathogen-free conditions. The sample size was divided into four cohorts, for the sake of manageability. Mice were between 5 and 9 weeks old at the beginning of the study, and BALB/cByJ mice were on average 8 days younger than C57BL/6J mice. Animals were individually identified using either tail marking reperformed every 2–3 days (cohort 1) or ear punching (cohort 2, 3 and 4) performed before beginning the study. The mice were housed in Type II polycarbonate cages (225 mm × 167 mm × 140 mm, cage floor area 375 cm^2^) in single sex and single strain groups of two (31 cages) to three (2 cages) animals. Trios rather than pairs happened when an extra mouse was provided by the breeding facility, which if not included would otherwise be euthanized, thus from an ethical and practical point-of-view were included in the sample. Each cage was provided with corncob bedding (LBS serving Biotechnology, UK), three sheets of absorbent paper (Renova, Portugal), half a cardboard tube (LBS serving Biotechnology, UK) for nesting and an amber-tinted acrylic tunnel (length 10 cm, diameter 5 cm), regardless of the assigned handling method. Cages were changed weekly by the person carrying out the experiments. Tunnels were disinfected every three weeks, to prevent frequent loss of home scent [30], and were wiped with absorbent paper before every weekly cage change. Food pellets (Teklad Harlan 2014S; Envigo, UK) and autoclaved tap water were provided ad libitum. Mice were housed under 12:12 h dark/light cycle, with lights on from 24:00 to 12:00, room temperature between 20–24 °C and 45–65% humidity. Scoresheets for general procedures in use at the i3S (which include weight monitoring) were filled out weekly. Due to aggression, a pair of male C57BL/6J mice had to be separated and housed individually for the last two days of the experiment. Monitoring of the ~3 mm wound after PIT-tag implantation was performed daily and, when necessary, iodine was applied on the area surrounding the wound, if scratched by animals during in the first days. All animals were handled by the same female experimenter during the testing.

### 2.2. Study Design

In this study we used two strains of mice, to enhance external validity of findings [31], as well as to allow comparisons between C57BL/6J, the most commonly used inbred strain of mice, and BALB/cByJ, a strain reportedly showing spontaneously elevated anxiety [32]. Despite the possibility of cage-effects from the potential impact of alarm calls between cage mates during handling [7], each animal was treated as an experimental unit, since all animals were handled individually, and the order by which they were handled was assigned randomly a priori, for each trial.

**Figure 1:**
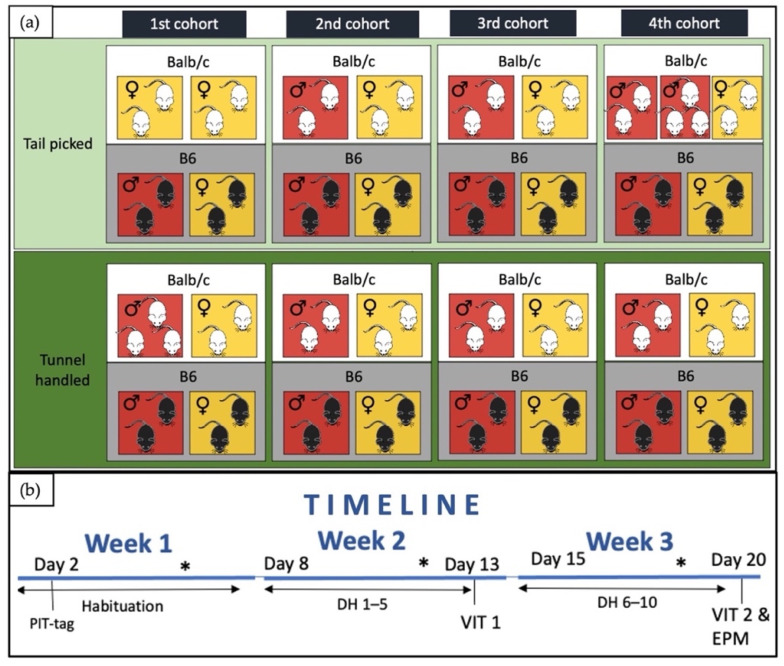
(**a**) Illustration of treatment arrangement for each cohort (showing inclusion of different strains (BALB/cByJ—Balc/c, white, C57BL/6J—B6, black), sexes (yellow—females, red—males)) and handling techniques (light green—tail-picked, dark green—tunnel-handled) and (**b**) timeline illustration of an experimental period for each cohort (PIT-tag—subcutaneous implantation of thermosensitive PIT-tag in the dorsal area under short general anesthesia; DH—daily handling for the duration of 10 days performed twice a day; VIT 1—1. trial of voluntary interaction test; VIT 2—2. trial of voluntary interaction test; EPM—elevated plus maze test; *—weekly weight measurement).

We planned our experiment using a factorial block design with strain, sex, handling method and drug treatment prior to EPM test as fixed factors, and each of the four cohorts as a block. Due to a breeding problem with BALB/cByJ mice at the animal facility, the experiment had to follow an incomplete random block design with 68 animals being divided into four cohorts as a block (1st cohort: 17 animals; 2nd and 3rd cohort: 16 animals; 4th cohort: 19 animals, Figure 1a). BALB/cByJ mice from the 2nd and 3rd cohort and all C57BL/6J mice were randomly assigned to all factors arranged within the cohort. The unbalance in treatment combinations in the 1st cohort was compensated for in the 4th cohort (Figure 1a).

Each cohort experiment was carried out over a period of three weeks (Figure 1b), with the first week serving as habituation to the tunnel, added on Day 1, and PIT-tag implantation, carried out on Day 2. Mice were subcutaneously implanted with a thermosensitive PIT-tag (Biomark^®^ Biotherm tags, 13 mm × 2.12 mm, glass-coated) in the dorsal area under short (<5 min) isoflurane anesthesia, using a syringe with 12 G needle, with the puncture site sealed by cyanoacrylate-based surgical glue (Vetbond^®^). From Day 8 to Day 12, mice were handled by their assigned handling technique twice a day during the light (9:00 h to 11:30 h) period and the dark (15:00 h to 17:30 h) period. This was followed by a voluntary interaction test (VIT) (1. trial), on Day 13 in the morning. The handling period was repeated with mice being handled with the assigned handling method from Day 15 to Day 19 twice a day during the light (9:00 h to 11:30 h) period and the dark (15:00 h to 17:30 h) period. On Day 20 mice were again tested by VIT (2. trial) in the morning, and by EPM in the afternoon.

### 2.3. Daily Handling (DH)

**Figure 2:**
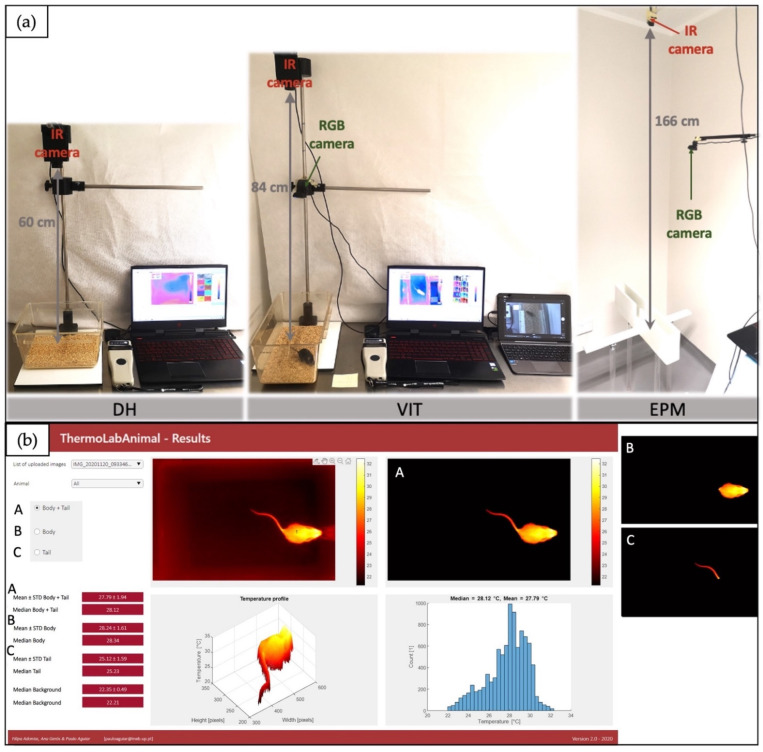
(**a**) Illustration of an experimental setting for Daily Handling (DH), Voluntary Interaction Test (VIT) and Elevated Plus Maze (EPM) and (**b**) output result from ThermoLabAnimal 2.0 software, using U-Net-based method, showing input image (upper left image), region recognized as animal (upper right image), with the possibility to separately analyze body and tail (**A**), only body (**B**) and only tail (**C**), with mean and median calculations provided for each region of interest.

The handling method (either tail or tunnel) was randomly assigned to each cage. Twice daily, animals were moved from the experimental room to the adjacent room, where the nesting material and tunnel were removed from the cage. Afterwards, each mouse was picked up using the assigned handling technique, as described by Hurst and West [27] (home cage tunnel was used for tunnel handled mice), and moved to an empty cage (Type II, 225 mm × 167 mm × 140 mm, cage floor area 375 cm^2^, with corncob bedding). We tested the animals in the DH trials for 2 min, as we were interested in the acute stress exposure, yet acknowledged that the exposure of 1 min, as the case in VIT, might not be sufficient to detect a significant physiological response. T_sc_ was measured with a PIT-tag reader at the beginning and the end of the test (0 s and 120 s), and IRT images, to measure T_body_ and T_tail_, were taken with a thermal camera (Thermal Expert EV1) placed 60 cm above the cage (Figure 2a) at time periods 0 s, 60 s and 120 s. Due to the non-invasiveness of IRT, thermal images were taken mid-test, which was not the case for PIT-tag readings, as these would likely impact the animals. Afterwards, the mouse was transported back to its home cage using the assigned handling technique, and the second mouse was assessed the same way. The same protocol was afterwards repeated with the third mouse in the two cases where cages housed trios. Testing order between the cages and within the cage was randomly assigned for each individual testing session. The assigned handling technique was also used during cage change or any other handling that mice were exposed to during the experiment.

### 2.4. Voluntary Interaction Test (VIT)

A VIT was performed on Day 13 and Day 20 in the morning (corresponding to the light period, in our facility), as described by Hurst and West [27], but with the following alterations: moving mice into a separate cage to obtain better IRT images, excluding 60 s habituation to the handler from the protocol as we were interested in the immediate appearance of SIH, and excluding the second repeat of the test. The duration of exposure to the handling equipment was kept to 1 min, as described in the original test. Mice were moved to the experimental room where nesting material and tunnel were removed from the cage. Each mouse was picked up using the assigned handling technique and moved to the test cage (Type II L, 325 mm × 170 mm × 140 mm, cage floor area 553 cm^2^, with corncob bedding) where the handling device was introduced for the duration of 60 s (either empty hand for tail-picked mice or hand holding a tunnel for tunnel-handled mice). The experimenter remained still in front of the cage during this period. T_sc_ was measured with PIT-tag reader at times 0 s and 60 s, and IR images, to measure T_body_ and T_tail_, were taken with the camera placed 84 cm above the cage (Figure 2a) at times 0 s, 30 s and 60 s. Afterwards, the first mouse was moved back to its home cage and the second and third mice were tested the same way. Visible light video was recorded with an RGB camera (LifeCam HD-3000) placed above the cage (Figure 2a), and videos were analyzed by an observer blinded to the treatments (with the exception of handling technique, which was observable from the video), to assess time spent with all four paws in the front half of the cage and time interacting with the handling device (sniffing the handling device, paws on, climbing on, chewing the glove or being inside the tunnel).

### 2.5. Elevated Plus Maze (EPM)

A single EPM test was performed on Day 20 in the afternoon (corresponding to the dark period, in our facility), as described by Walf and Frye [33]. Half of the animals received an IP injection of diazepam (1.5 mg/kg, a dose that significantly impacts anxiety-related behaviour in EPM of both mice strains [29]) while the other half received an IP injection of a saline solution, 30 min before the test. Drug treatment was randomly assigned to individuals within the same cage, and the allocation of treatment was concealed from the experimenter. After injection, animals were moved to the experimental room for habituation period, 30 min prior to the test. Nesting material and tunnel were removed before the beginning of the test. At the start of the test, the mouse was picked up using the assigned handling method and placed in the center of a grey colored wooden EPM apparatus (elevated 53 cm, arms 29.5 cm × 6.5 cm, wall 14.5 cm), facing an open arm. The experimenter was hidden from view, during the 5 min duration of the test. T_sc_ was measured with a PIT-tag reader at times 0 s and 300 s, and IR images to measure T_body_, were taken with a camera placed 166 cm above the apparatus (Figure 2a) at times 0 s, 30 s, 60 s, 90 s, 120 s, 150 s, 180 s, 210 s, 240 s, 270 s and 300 s. After the test, the mouse was moved to a separate closed cage to minimize the impact of sending olfactory and sound signals to its cage-mate that was being tested in the same method as described above. Between each animal the EPM was cleaned with 70% alcohol. Each test was video recorded with an RGB camera placed above the apparatus (Figure 2a), and videos were analyzed by an observer blinded to the treatments, to assess time spent in open and closed arms with all four paws and the number of entries in the open and closed arm.

### 2.6. Equipment and Software

Thermosensitive PIT-tags (Biotherm13) were read using a Biomark^®^ GPR Plus reader (temperature range 33 to 43 °C), with a reading distance of around 5 cm, and a ‘Thermal Expert’ TE-EV1 thermal camera (640 × 480 resolution, 19mm lens with 32° × 24° (40° diagonal) angle, thermal sensitivity ≤50 mK, switched on until readings stabilized before being used, which took from 30 to 60 min) used for IRT images. Images were analyzed using in-house developed ThermoLabAnimal 2.0 software (Figure 2b, Appendix A), which performed automatic segmentation with one of two options: by Otsu method (to detect mice using an automatic temperature threshold level), or by a modified U-Net-based method (using a deep learning network that was previously trained to identify mice and segment them into body and tail region).

Mean temperature is calculated based on pixels recognized as region of interest. Unless stated otherwise, the region of interest corresponds to the result of the automatic segmentation operation. With the U-Net-based method, tail and body are automatically segmented and the corresponding mean temperatures are extracted. With the Otsu method, for the tail mean temperature the user needs to define a coarse sub-region containing the tail and excluding the body (no requirement for fine delineation). Images obtained during DH and VIT were analyzed using the U-Net-based method, while EPM images, due to poor resolution (the camera had to be placed far above the EPM apparatus, to capture it entirely), were analyzed using the Otsu method (tail temperature not assessed). LifeCam HD-3000 was used to obtain videos for behavioural analysis. BORIS software was used for video analysis.

For each time-point, three IR images were taken. Images were then individually selected to exclude those where the full body was not visible because animals were rearing up or entering the tunnel during VIT and average temperature from all images taken for each time-point was calculated.

### 2.7. Analysis and Statistics

The research protocol did not allow allocation concealment of the treatment. However, the researcher analyzing the data was blinded to the treatments. Sample size was calculated to detect an effect size of at least Cohen’s f = 0.35 (partial eta square ηp^2^ ≈ 0.11) with 80% power, for α = 0.05, which Wahlsten [34] proposes as medium effect sizes for inbred animals in a controlled environment.

All statistical tests were performed using SPSS (version 27.0). A repeated-measures ANOVA was performed to analyze temperature variation during behavioural tests, with handling technique, strain and sex (plus light/dark period for DH, and drug treatment for EPM) as fixed factors. Day of trial was analyzed separately for DH, and likewise for the cohort. When the sphericity condition was not met (Mauchly’s test), Greenhouse–Geisser correction was used. Šidák method was used for pairwise comparison correction, as well as for multiple comparisons correction. We serendipitously confirmed previous observations [6,7] that, within each cage, animals that tested second consistently had higher body temperatures than animals tested first. Hence, we ran statistical tests to verify whether the said difference was significant and consistent across sexes, strains and drug treatments (all treated as fixed factors in repeated-measures ANOVA), even though it had not been included in the preregistered hypotheses, since testing order had been randomly assigned. Due to the low occurrence of animals tested third in VIT and EPM (only two cages with mice housed in trios, in two trials for VIT and one trial for EPM, whereas each animal went through 20 trials of DH), animals tested third were included only in the analysis for DH, and excluded from VIT and EPM, when analyzing for the impact of testing order within the cage. Univariate ANOVA was performed for analysis of performance in behavioural tests, with handling technique, strain, sex, trial for VIT, and drug treatment for EPM as fixed factors, and cohort as random factor. The aforementioned Šidák corrections were applied. Graphs were created using GraphPad 6.0.

## 3. Results

Table 1 represents the summary of our results in response to the previously established hypotheses.

### 3.1. Daily Handling (DH) Effects on Temperature

**Figure 3:**
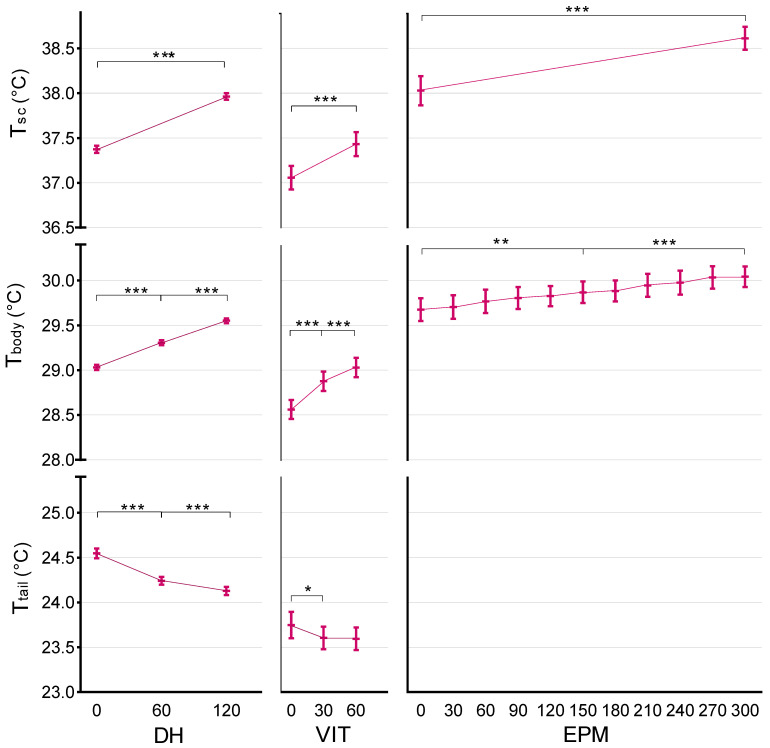
T_sc_, T_body_ and T_tail_ during Daily Handling (DH), Voluntary Interaction Test (VIT) and Elevated Plus Maze (EPM), at predefined time points, presented for all animals. Errors bars represent 95% CI, * *p* < 0.05, ** *p* < 0.01, *** *p* < 0.001.

**Figure 4:**
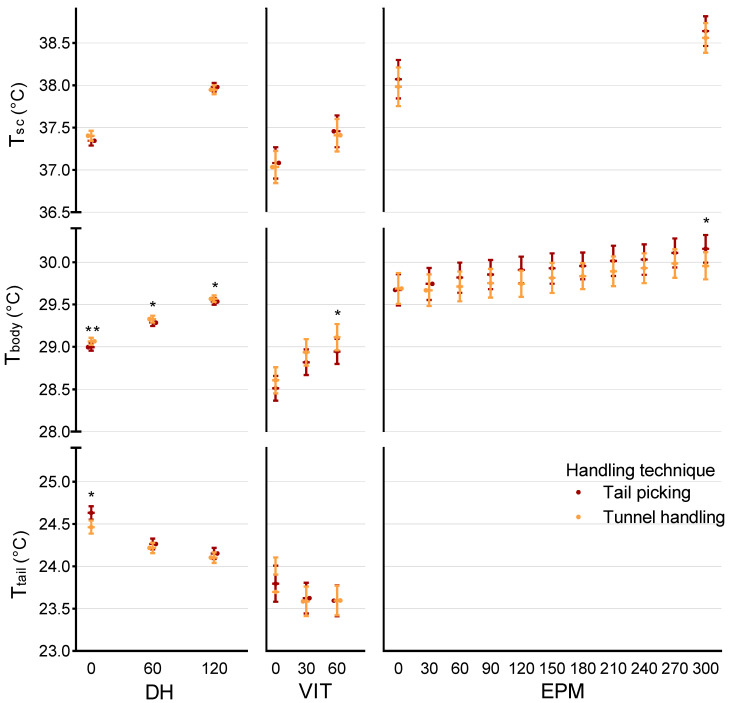
T_sc_, T_body_ and T_tail_ during Daily Handling (DH), Voluntary Interaction Test (VIT) and Elevated Plus Maze (EPM), at predefined time points, presented for effect of handling technique (orange—tunnel-handled, red—tail-picked). Errors bars represent 95% CI, * *p* < 0.05, ** *p* < 0.01.

In the 2 min daily handling (DH), after the mice were picked up and placed in another cage, T_sc_ rose significantly (F = 5923.05, *p* < 0.001, Figure 3, DH-T_sc_) from $\bar{x}$ = 37.37 °C (95% CI (37.33, 37.41)) at 0 s to $\bar{x}$ = 37.96 °C (95% CI (37.92, 38.00)) at 120 s. This temperature rise was observable across sexes, strains, handling techniques, time of light/dark cycle, and cohorts, with no differences found between the daily trials. No differences were observed between animals handled with different techniques (Figure 4, DH-T_sc_).

Strain differences were significant (F = 16.62, *p* < 0.001), with BALB/cByJ mice showing higher T_sc_ at 0 s ($\bar{x}$ = 37.48 °C, 95% CI (37.43, 37.54)) and 120 s ($\bar{x}$ = 38.00 °C, 95% CI (37.96, 38.06)) than C57BL/6J mice (at 0 s $\bar{x}$ = 37.26 °C, 95% CI (37.20, 37.32) and at 120 s $\bar{x}$ = 37.91 °C, 95% CI (37.86, 37.97)). Sex had a small albeit statistically significant effect on T_sc_ (F = 5.29, *p* = 0.004), with males showing higher temperature (at 0 s $\bar{x}$ = 37.37 °C, 95% CI (37.32, 37.42) and at 120 s $\bar{x}$ = 38.01 °C, 95% CI (37.96, 38.06)), than females (at 0 s $\bar{x}$ = 37.33 °C, 95% CI (37.28, 37.38) and at 120 s $\bar{x}$ = 37.87 °C, 95% CI (37.83, 37.92)). T_sc_ was also significantly higher (F = 156.13, *p* < 0.001) in the dark period than in the light period, on average $\bar{x}$ = +0.49 °C (95% CI (0.41, 0.57)). A sex * handling technique interaction (F = 12.18, *p* < 0.001) was observed for T_sc_, with female tail-handled mice showing a higher T_sc_ increase, whereas in males, it was tunnel-handled mice that showed a higher increase in T_sc_. A strain * handling technique interaction (F = 7.57, *p* = 0.006) was also present, with tail-picked C57BL/6J having higher T_sc_, while tail-picked BALB/cByJ had lower T_sc_ during DH.

Similar to T_sc_, infrared thermography-assessed T_body_ rose significantly during DH (F = 3034.30, *p* < 0.001, Figure 3, DH-T_body_) from $\bar{x}$ = 29.03 °C (95% CI (29.00, 29.06)) at 0 s, to $\bar{x}$ = 29.31 °C (95%CI (29.28, 29.34)) at 60 s, reaching $\bar{x}$ = 29.55 °C (95% CI (29.52, 29.58)) at 120 s. Basal T_body_ differed significantly (F = 71.75, *p* < 0.001) between all cohorts, and in all of them temperature rose during DH. T_body_ was marginally yet significantly (F = 6.17, *p* = 0.013) higher for male mice than female mice across all three time-points ($\bar{x}$ = +0.07 °C, 95% CI (0.15, 0.13)), and likewise (F = 6.38, *p* = 0.012, Figure 4, DH-T_body_) for tunnel-handled mice as compared to tail-picked mice ($\bar{x}$ = +0.07 °C, 95% CI (0.17, 0.13)). Similar to T_sc_, T_body_ was significantly (F = 36.61, *p* < 0.001) higher during the dark period (on average $\bar{x}$ = +0.17 °C, 95% CI (0.12, 0.23)) than in the light period. No T_body_ differences were found between strains and day of testing.

T_tail_ dropped significantly during DH trials (F = 256.81, *p* < 0.001, Figure 3, DH-T_tail_) from $\bar{x}$ = 24.55 °C (95% CI (24.49, 24.60)) at 0 s to $\bar{x}$ = 24.24 °C (95% CI (24.12, 24.29)) at 60 s and was lowest ($\bar{x}$ = 24.13 °C, 95% CI (24.08, 24.18)) at 120 s. Only sex was found to have a significant effect (F = 8.13, *p* = 0.004); though no sex differences were observable at 0 s (both averaging  = 24.55 °C), T_tail_ became increasingly lower for female mice at 60 s ( = 24.17 °C, 95% CI (24.10, 24.23) and at 120 s ($\bar{x}$ = 24.00 °C, 95% CI (23.94, 24.07)) as compared to male mice (at 60 s $\bar{x}$ = 24.31 °C, 95%CI (24.25, 24.38) and at 120 s $\bar{x}$ = 24.25 °C, 95% CI (24.18, 24.31)).

### 3.2. Voluntary Interaction Test (VIT) Effects on Temperature

T_sc_ rose significantly during 1 min exposure to VIT (F = 136.93, *p* < 0.001, Figure 3, VIT-T_sc_) from $\bar{x}$ = 37.06 °C (95% CI (36.92, 37.19)) at 0 s, to $\bar{x}$ = 37.43 °C (95% CI (37.29, 37.57)) at 120 s, with no significant differences between the two trials. Strain was the only fixed factor found to have an effect on T_sc_ in the VIT test, with BALB/cByJ mice showing significantly (F = 6.32, *p* = 0.013) higher T_sc_ than C57BL/6J (on average $\bar{x}$ = 0.34, 95% CI (0.07, 0.61)).

T_body_ rose significantly (F = 136.93, *p* < 0.001, Figure 3, VIT-T_body_) during VIT, from $\bar{x}$ = 28.56 °C (95% CI (28.45, 28.67)) at 0 s, to $\bar{x}$ = 28.88 °C (95% CI (28.77, 28.99)) at 30 s and was highest at 60 s ($\bar{x}$ = 29.03 °C, 95% CI (28.92, 29.14)). None of the factors under study were found to have a significant effect, which was also observable for T_tail_, which dropped slightly, but significantly (F = 5.84, *p* = 0.004, Figure 3, VIT-T_tail_) in the VIT, from 0 s ($\bar{x}$ = 23.76 °C, 95% CI (23.62, 23.91)) to 30 s ($\bar{x}$ = 23.62 °C, 95% CI (23.50, 23.75)), remaining stable until 60 s ($\bar{x}$ = 23.60 °C, 95% CI (23.47, 23.73)).

### 3.3. Elevated Plus Maze (EPM) Effects on Temperature

As expected, T_sc_ rose significantly (F = 93.91, *p* < 0.001, Figure 3, EPM-T_sc_) in the EPM from 0 to 300 s on average $\bar{x}$ = +0.57 °C (95% CI (0.45, 0.69)). An overall significant difference was found between strains (F = 6.91, *p* = 0.011), with higher T_sc_ for BALB/cByJ ($\bar{x}$ = 38.29 °C, 95% CI (37.08, 38.50) at 0 s and $\bar{x}$ = 38.67 °C, 95% CI (38.50, 38.84) at 300 s) than for C57BL/6J mice ($\bar{x}$ = 37.76 °C, 95%CI (37.53, 37.99) at 0 s and $\bar{x}$ = 38.52 °C, 95% CI (38.34, 38.71) at 300 s), and sexes (F = 5.59, *p* = 0.022), with males showing higher T_sc_ ($\bar{x}$ = 38.16 °C, 95% CI (37.94, 38.38) at 0 s and $\bar{x}$ = 38.77 °C, 95% CI (38.59, 38.95) at 300 s) than for females ($\bar{x}$ = 37.89 °C, 95% CI (37.68, 38.11) at 0 s and $\bar{x}$ = 38.42 °C, 95% CI (38.25, 38.60) at 300 s). Drugs were observed to have a statistically significant effect on T_sc_ only at 0 s (significant drug * time interaction, F = 12.81, *p* = 0.001) with diazepam-treated mice showing lower temperature ($\bar{x}$ = 37.81 °C 95% CI (37.59, 38.03)) than vehicle-treated mice ($\bar{x}$ = 38.24 °C 95% CI (38.03, 38.46)), since at 300 s the diazepam-treated mice had reached virtually the same temperature ($\bar{x}$ = 38.59 °C, 95% CI (38.41, 38.77)) of vehicle-treated mice ($\bar{x}$ = 38.60 °C, 95%CI (38.43, 38.77)).

T_body_ showed a significant (F = 23.89, *p* < 0.001, Figure 3, EPM-T_body_) increase during EPM ($\bar{x}$ = +0.38 °C, 95% CI (0.20, 0.55)). Among the variables of interest, only drug treatment had an effect on T_body_ during EPM (F = 11.21, *p* = 0.02, Figure 6a), with vehicle-treated mice showing overall higher temperature (at 0 s $\bar{x}$ = 29.90 °C, 95% CI (29.72, 30.07) and at 300 s $\bar{x}$ = 30.15 °C, 95% CI (30.00, 30.31)), than diazepam-treated mice (at 0 s $\bar{x}$ = 29.47 °C, 95% CI (29.30, 29.64) and at 300 s $\bar{x}$ = 29.97 °C, 95% CI (29.81, 30.12)).

### 3.4. Behaviour in the Voluntary Interaction Test (VIT)

**Figure 5:**
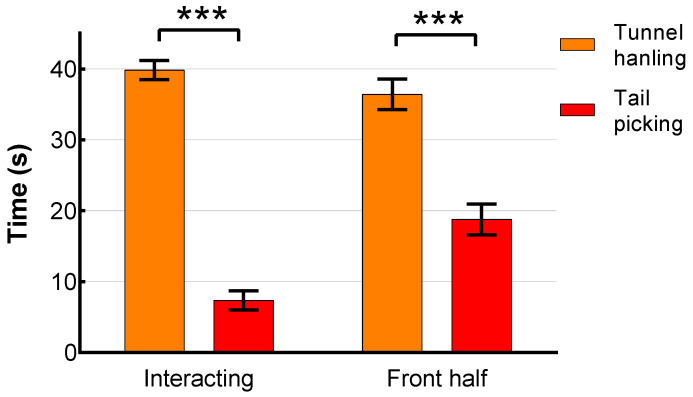
Time spent in front half of the cage and time interacting with the handler during 1 min Voluntary Interaction Test (VIT), separated for handling technique (orange—tunnel-handled, red—tail-picked). Errors bars represent 95% CI, *** *p* < 0.001.

There was a statistically significant effect of handling technique (F = 136.28, *p* < 0.001, Figure 5, front half) in time spent in the front half of the cage (closer to handling equipment and handler), with tunnel-handled mice spending on average almost twice as long in the front half next to the handling apparatus ($\bar{x}$ = 36.45 s, 95% CI (34.31, 38.59)) than tail-picked mice ($\bar{x}$ = 18.76 s, 95% CI (16.69, 20.84)). Strain was also found to have an effect (F = 9.22, *p* = 0.003), with BALB/cByJ mice spending more time in the front half of the cage ($\bar{x}$ = 29.90 s, 95% CI (27.84, 31.96)) than C57BL/6J ($\bar{x}$ = 25.31 s, 95% CI (23.14, 27.41)). However, this difference was possibly due to handling technique (handling technique * strain significant interaction, F = 4.00, *p* = 0.048), since no strain differences in time spent near the handling device were observed for tail-picked mice. A handling technique * sex interaction was also found (F = 10.67, *p* = 0.001), with tail-picked males spending more time ($\bar{x}$ = 21.50 s, 95% CI (18.46, 24.55)) near the handler’s empty hand than females ($\bar{x}$ = 16.02 s, 95% CI (13.10, 18.95)), whereas tunnel-handled females spent more time ($\bar{x}$ = 38.70 s, 95% CI (35.63, 41.77)) near handling equipment than tunnel-handled males ($\bar{x}$ = 34.20 s, 95% CI (31.21, 37.16)).

Time spent interacting directly with the handling apparatus (i.e., tail-picked mice interacting with the hand and tunnel-handled mice interacting with hand holding a tunnel) also differed significantly between handling techniques (F = 1149.66, *p* < 0.001, Figure 5, Interacting). Tunnel-handled mice interacted more than five times longer with the handling equipment ($\bar{x}$ = 39.87 s, 95% CI (38.50, 41.24)) than tail-picked mice ($\bar{x}$ = 7.01 s, 95% CI (5.67, 8.36)).

### 3.5. Behaviour in the Elevated Plus Maze (EPM)

**Figure 6:**
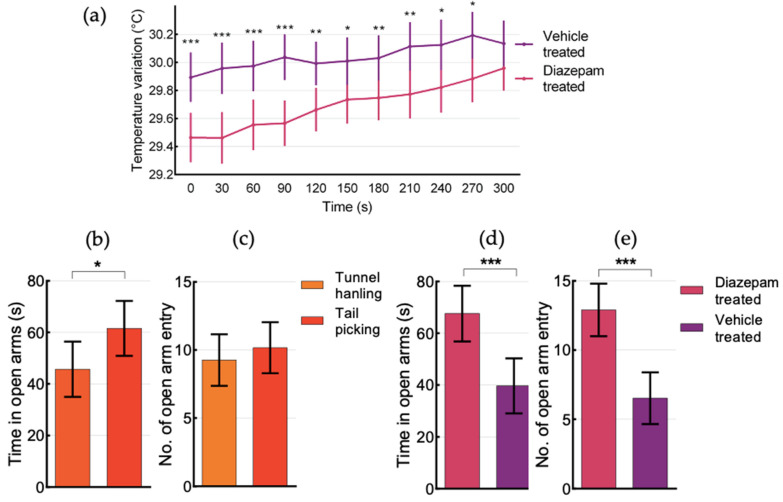
Elevated Plus Maze (EPM) differences for (**a**) T_body_ between diazepam- and vehicle-treated mice (**b**) time spent in open arms and (**c**) number of open arm entries between tunnel-handled and tail-picked mice, (**d**) time spent in open arms and (**e**) number of open arm entries between diazepam- and vehicle-treated mice. Error bars represent 95% CI, * *p* < 0.05, ** *p* < 0.01, *** *p* < 0.001.

Tail-picked mice spent significantly (F = 4.62, *p* = 0.036, Figure 6b) more time in the open arms ($\bar{x}$ = 62.16 s, 95% CI (51.62, 72.71)) than tunnel-handled mice ($\bar{x}$ = 46.13 s, 95% CI (35.54, 56.72)), while there was no difference in the number of open arm entries (Figure 6c). BALB/cByJ mice spent significantly (F = 6.01, *p* = 0.018) more time in the open arms ($\bar{x}$ = 63.29 s, 95% CI (53.10, 73.49)) than C57BL/6J mice ($\bar{x}$ = 45.00 s, 95% CI (34.06, 59.94)). Diazepam treatment increased time spent in open arms (F = 14.07, *p* < 0.001, Figure 6d), with diazepam treated mice spending $\bar{x}$ = 68.11 s (95% CI (57.46, 78.77)) in open arms, while vehicle treated mice spent $\bar{x}$ = 40.18 s (95% CI (29.71, 50.65)) in open arms. Diazepam-treated mice also had significantly (F = 22.06, *p* < 0.001, Figure 6e) more entries into open arms ($\bar{x}$ = 12.96, 95% CI (10.99, 14.92)) than vehicle-treated mice ($\bar{x}$ = 6.50, 95% CI (4.57, 8.43)).

### 3.6. Effect of Testing Order

**Figure 7:**
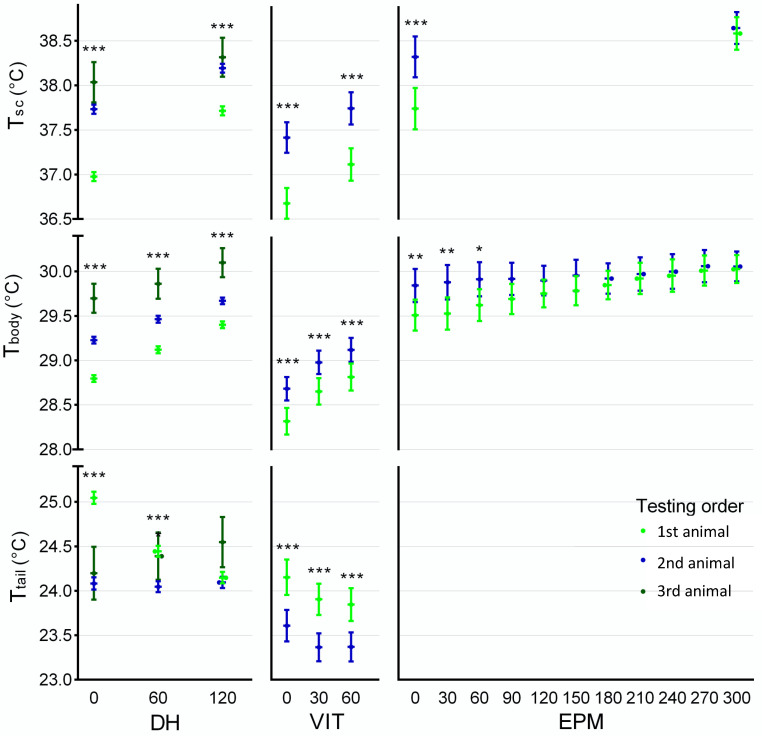
T_sc_, T_body_ and T_tail_ during Daily Handling (DH), Voluntary Interaction Test (VIT) and Elevated Plus Maze (EPM), at predefined time points, presented for effect of testing order (1st animal from cage tested—light green, 2nd animal from cage tested—blue, 3rd animal from cage tested—dark green). Error bars represent 95% CI, * *p* < 0.05, ** *p* < 0.01, *** *p* < 0.001.

During DH, testing order had a significant effect on T_sc_ (F = 134.63, *p* < 0.001, Figure 7, DH-T_sc_), with T_sc_ being higher for animals tested second as compared to animals tested first and higher still for animals tested third, regardless of strain, sex or handling technique. The mean difference at 0 s was on average $\bar{x}$ = +0.75 °C (95% CI (0.72, 0.76), *p* < 0.001) between mice tested first and mice tested second and on average $\bar{x}$ = +0.30 °C (95% CI (0.02, 0.58), *p* = 0.028) between the latter and mice tested third, for the cages housing trios. At 120 s, differences (F = 79.55, *p* < 0.001) were significant between animals tested first and second, but not between the latter and those tested third (when applicable).

For DH, T_body_ followed the same significant (F = 100.05, *p* < 0.001, Figure 7, DH-T_body_) rising trend, with mean average differences between animals tested first and second $\bar{x}$ = +0.44 °C (95% CI (0.37, 0.51), *p* < 0.001) and between second and third $\bar{x}$ = +0.49 °C (95% CI (0.29, 0.69), *p* < 0.001). These were, respectively, $\bar{x}$ = +0.35 °C (95% CI (0.28, 0.42), *p* < 0.001) and $\bar{x}$ = +0.40 °C (95% CI (0.19, 0.61), *p* < 0.001) at 60 s and, also, respectively, $\bar{x}$ = +0.28 °C (95% CI (0.21, 0.35), *p* < 0.001) and $\bar{x}$ = +0.46 °C (95% CI (0.26, 0.66), *p* < 0.001) at 120 s.

Testing order significantly affected T_tail_ (F = 60.43, *p* < 0.001, Figure 7, DH-T_tail_) across all groups. At 0 s the mean difference between animals tested first and second was $\bar{x}$ = −0.97 °C (95% CI (−1.1, −0.83)), while it was narrower at 60 s ($\bar{x}$ = 0.40 °C, 95% CI (−0.53, 0.28)) and almost indistinguishable at 120 s ($\bar{x}$ = +0.04 °C, 95% CI (−0.19, +0.07)) as animals tested second had already reached the lowest T_tail_ at the start of the test, which the animals tested first reached at 120 s. In the cases where a third animal was present, we observed their T_tail_ at 0 s was lower than that of animals tested first, and then rose further until 120 s, becoming at that point higher than both animals tested first or second.

Moreover, the testing order was found to have a significant impact during the VIT, across sexes, strains, and handling technique. The second mouse tested showed higher T_sc_ (F = 29.75, *p* < 0.001, Figure 7, VIT-T_sc_) at both 0 s and 60 s (on average $\bar{x}$ = +0.69 °C, 95% CI (0.44, 0.93)), and the same was observable for T_body_ (F = 11.45, *p* = 0.01, Figure 7, VIT-T_body_), with average increase $\bar{x}$ = +0.33 °C (95% CI (0.14, 0.52)), also across all three time-points (0 s, 30 s and 60 s) and groups. Similar to DH, in the VIT T_tail_ was significantly (F = 25.01, *p* < 0.001, Figure 7, VIT-T_tail_) lower in mice tested in second place ($\bar{x}$ = −0.60 °C 95% CI (−0.82, −0.38), consistently across sexes, strains, and handling technique.

For T_sc_ during EPM, testing order had a significant effect (F = 16.04, *p* < 0.001, Figure 7, EPM-T_sc_), though animals tested second had a higher temperature only at 0 s ($\bar{x}$ = +0.56, C, 95% CI (0.28, 0.82)), since at 300 s no differences were observable. There was a significant testing order * drug interaction (F = 10.15, *p* = 0.003), as diazepam-treated animals showed no differences between animals tested first and second, neither at 0 s nor 300 s. The same was observed for T_body_ (F = 4.11, *p* = 0.048, Figure 7, EPM-T_body_), with the second mouse tested showing significantly a higher temperature at 0 s (F = 10.88, *p* = 0.002) and up until 60 s, but not from 90 s to 300 s. Likewise to T_sc_, there was a drug * testing order interaction (F = 10.15, *p* = 0.003), as testing order had no impact on the hyperthermic stress response in the EPM in diazepam-treated animals, whereas in vehicle-treated animals T_body_ in animals tested second remained higher ($\bar{x}$ = +0.58 °C, 95% CI (0.25, 0.91), F = 13.60, *p* = 0.002, ) from start to finish of the EPM.

## 4. Discussion

In this study, we compared body surface temperatures measured by infrared thermography (IRT) with temperatures from subcutaneously-implanted thermosensitive PIT-tags, during exposure to three different mild stressors, Daily Handling (DH), Voluntary Interaction Test (VIT) and the Elevated Plus Maze (EPM). While duration of the tests varied between 1 and 5 min, stressed-induced hyperthermia (SIH) responses were consistent between the three. They were characterized by a quick-onset of rise in body temperature (measured by reading of subcutaneous thermosensitive PIT tags—T_sc_) and mean body surface temperature (assessed by infrared thermography—T_body_), as a reflection of vasodilation of blood flow to skeletal muscles and brain [3,17] preparing the animal for “fight or flight” [17]. This was observed concomitant with a decrease in tail temperature (assessed by infrared thermography—T_tail_), resulting from vasoconstriction of blood flow to the tail, as the tail plays an important role in thermoregulation of rodents [35], while vasoconstriction is also believed to prevent extensive bleeding in case of injury [17]. In this regard, our findings are in agreement with previous research on SIH in rodents [15,19,36,37], in response to all mild stressors tested, regardless of duration. However, both T_body_ and T_sc_ rose for the whole duration of the 5 min EPM test, a trend that had not plateaued at 300 s, showing that the time the animal is exposed to the stressor, has an important effect on the body temperature increase. Vianna and Carrive [15], measuring maximum temperature of a shaved skin region, observed that different body surface regions reach maximum temperature 4 to 8 min after exposure to the open field, an increase ranging between 2.0 and 3.4 °C. Duparcq et al., [37] observed that the maximum eye temperature peaked within 1 min, while tail temperature dropped to its lowest at around 2 min after exposure to a novel environment. Drop in tail temperature lasting for at least 2 min was observed also in our study, with this short-term vasoconstriction resulting in reduction of heat dissipated through the tail surface [22]. This initial drop in temperature can, during prolonged stress exposure, be followed by an increase in tail temperature [17], as a means to dissipate excess heat [22]. We believe that the increase in T_tail_ of the third animal tested during the DH test provides support for this explanation. Duparcq et al., [37] found a maximum peripheral body temperature (usually in the eye) rise of 0.65 °C and tail temperature drop of 0.25 to 0.64 °C. In our study, we used mean body surface temperature, rather than maximum temperature, which prevents a direct comparison with the aforementioned results, which moreover focused on specific regions. Nonetheless, all studies showed that a short-onset SIH response can be observed as early as 30 to 60 s. Moreover, we observed SIH response to be more consistent and reliable in longer (2 to 5 min) periods of stress exposure.

Measurable physiological differences between the two mouse strains used have been reported [38], with BALB/cByJ also being reportedly more prone to spontaneously elevated anxiety behaviour during behavioural tests [32]. Therefore, it was expected that mice of this strain would have a higher temperature rise in response to a stressor. However, while BALB/cByJ showed modest but significantly higher T_sc_ than C57BL/6J in all tests, this was not observable for T_body_. It should be noted that due to breeding problems in our facility BALB/cByJ were on average eight days younger than C57BL/6J animals, and while not likely to have a meaningful impact, we consider it should be reported as a possible source of bias. Recently, Faraji and Metz [39] reported sex differences in thermal response in mice. In our study, male mice showed modest yet significantly higher T_sc_ temperatures during DH and EPM, though not found to be significant for T_body_. Interestingly, T_tail_ dropped to lower values in females during DH. 

Although tail picking has been amply reported to cause more stress to laboratory mice when compared to tunnel handling [27,28], we did not detect a robust and consistent difference in T_sc_, T_body_ or T_tail_ between tail-picked and tunnel-handled mice. It is possible that stress from cage manipulation masked putative handling method differences in our experiment. Prior to handling, mice were first moved to an experimental room, where tunnel and nesting material were removed from the cage, and only then were they removed from the cage using the assigned handling technique. It has been reported that moving the cages [40,41] and exposing rodents to a novel environment [15,37] is in itself stressful, therefore this could have enough of an impact to increase their temperature [38]. Both our tail-picked and tunnel-handled mice were exposed to only short 1 to 2 s handling periods, reported by Gouveia and Hurst [28] to be sufficient in bearing a measurable effect, but in our experimental context the overall experience of the mice may have overshadowed it. If so, SIH would not be recommended as a measure of such low-grade stress differences, or at least in the context of other already stressful situations.

Testing order had a significant and robust effect across all groups and tests, as mice that were the second or third to be tested had higher temperatures than the first mouse to be tested in a cage. Whereas we did not have a prior hypothesis about test order effect, the study was designed to fully randomize testing order within cages, for all tests and trials. Therefore, we believe that the testing order can be analyzed post hoc without falling into the pitfall of HARKing [42]. The finding of higher temperature in animals tested later corroborates previous reports by Zethof et al. [6] and Borsini et al. [43], as mice that were removed from the cage later showed higher rectal temperature, as compared to those removed first. The fact that the only case where we did not observe a testing order effect was in diazepam-treated mice, strongly suggests that the heightened thermal response results from stress and anxiety building up in mice expecting to be handled. This is furthermore validated by the lower tail temperatures in these animals. To further verify if the temperature increase is a result of anxiety, rather than increased activity, we additionally analyzed the activity level (distance and speed travelled) in the EPM (Appendix B). In fact, we confirmed that diazepam-treated animals were more active, while showing lower temperature, confirming our hypothesis that we indeed observed anxiety related temperature increase. While cages were closed during the trials of the first animal to minimize the potential impact of any alarm calls to the second animal, as was reported could be the case by Zethof [6], the stress of waiting in social isolation without nesting material or a hiding place for a longer period may account for the robust differences observed. If that is so, social isolation, combined with environmental disturbance and anticipatory stress (i.e., anxiety), even in the home cage, plays a more relevant role than we had anticipated, and highlights the importance to control for the order of testing in behavioural studies in mice.

During the VIT, we did not observe a handling technique difference for temperature variation. This difference was, however, quite striking in the behaviour parameters themselves, as tunnel-handled mice were much more prone to interact with the handler and spend more time in the front half of the cage, as compared to tail-picked mice. However, there may be a methodological bias resulting from using a tunnel in the VIT for tunnel-handled animals (as the test originally described by [27]), rather than a hand, since it is recognized as a familiar object in an unfamiliar and uncomfortable situation (novel barren cage, separation from cage mate, bright light, presence of handler) and may moreover be perceived as a refuge for these thigmotaxic animals. Nevertheless, Henderson et al. [44] exposed both handling groups to a hand without the tunnel during the VIT, and came to the same results that tunnel-handled mice are more likely to interact with the handler.

During EPM testing we found no differences between the handling groups for either temperature variations or behavioural results. While this is in contrast with findings from Hurst and West [27] and Clarkson et al. [45], it agrees with results from Nakamura and Suzuki [46] who also did not observe differences between handling techniques in the EPM. These conflicting results raise the question of whether tail picking is markedly more anxiogenic than tunnel handling, at least in our study. The method of tail picking used in these studies does not involve literally suspending animals by the tail for any amount of time, but rather gently pulling them by the base of the tail to the back of the handler’s hand and moving them using this method to another cage. This is arguably a more refined approach to actually lifting mice by the tail and may plausibly account to some extent for a lower effect size than our sample size would allow detection of.

The use of IRT, given its non-invasiveness, allows high temporal resolution of measurements. For the analysis of the thermal images, we used the in-house developed ThermoLabAnimal 2.0, which expands on the functionalities of its predecessor [47]. Its use of a machine-learning algorithm allows obtaining objective results without the operator-induced errors and variability expected from manual region-of-interest definition, while segmenting body and tail and presenting thermal data separately for each part.

Overall, we believe our results validate IRT as a non-invasive means to assess stress/anxiety by measuring SIH. This is grounded on: (a) the observed thermal response to a range of mild stressors, (b) the mitigation of SIH in anxiolytic-treated animals as compared to vehicle controls, (c) drop in tail temperature concomitant with T_sc_ and T_body_ (a good indicator of valence of the response), (d) agreement between behavioural and physiological effects of anxiolytic-treatment, and (e) the observable impact of anticipatory stress in SIH, from a comparison of cage-mates tested first and second.

## 5. Conclusions

IRT is a non-invasive method in tracking temperature changes in rodents and to assess stress responses. The ability to non-invasively assess body and tail temperatures in rodents is an added advantage of this approach, given that the tail temperature initially varies in the opposite direction of the rest of the body, during acute stress exposure. While differences in SIH were not found to be significant between animals handled by different methods, we were however able to identify the impact and intensity of other different stressors.

## Figures and Tables

**Table 1:** Summary of the results in relation to the specific hypotheses (stress-induced hyperthermia (SIH), infrared thermography (IRT), temperature from subcutaneously-implanted thermosensitive PIT-tags (T_sc_), mean body surface temperature measured with IRT (T_body_), mean tail surface temperature measured with IRT (T_tail_), Daily Handling (DH), Voluntary Interaction Test (VIT) and Elevated Plus Maze (EPM)).

Hypotheses	Confirmed	Evidence	
I IRT can detect hyperthermic stress response in mice	Yes	DH	Average T_body_ rise of 0.5 °C (*p* < 0.001) and T_tail_ drop of 0.4 °C (*p* < 0.001).
		VIT	Average T_body_ rise of 0.5 °C (*p* < 0.001) and T_tail_ drop of 0.2 °C (*p* = 0.004).
		EPM	Average T_body_ rise of 0.4 °C (*p* < 0.001).
II Hyperthermic stress responses differ between tail-picked and tunnel-handled animals	No	DH	T_body_ higher (*p* = 0.012) in tunnel-handled mice. No handling technique differences in T_sc_ or T_tail_.
		VIT	No handling technique differences in T_sc_, T_body_ or T_tail_.
		EPM	No handling technique differences in T_sc_ or T_body_.
III Findings will be consistent between the two sexes	Mostly	DH	Small yet significant sex differences in T_sc_ (*p* = 0.004), T_body_ (*p* = 0.013) and T_tail_ (*p* = 0.004).
		VIT	No sex differences in T_sc_, T_body_ or T_tail_.
		EPM	No sex differences in T_sc_ or T_body_.
Findings will be consistent between the two mouse strains	Yes (T_tail_, T_body_) No (T_sc_)	DH	BALB/cByJ show higher (*p* < 0.001) T_sc_. No strain differences in T_body_ and T_tail_.
		VIT	BALB/cByJ show higher (*p* = 0.013) T_sc_. No strain differences in T_body_ and T_tail_.
		EPM	BALB/cByJ show higher (*p* = 0.011) T_sc_. No strain differences in T_body_ and T_tail_.
IV T_body_ and T_tail_ will be consistent with T_sc_	Mostly	Both T_sc_ and T_body_ rose during all behavioural tests and were higher during the dark period. Effect of testing order were observed in both T_sc_ and T_body_. No consistent differences detected between the two strains of mice.	
V Magnitude of SIH indicates stress intensity, in accordance with behavioural test	Not clear	VIT was able to detect differences between the two handling techniques (*p* < 0.001). Handling technique differences were not observed during EPM or in thermal response (T_sc_, T_body_ and T_tail_). Further research needed to test the magnitude intensity for which SIH can be detected.	
IV SIH in EPM will be less pronounced in animals treated with an anxiolytic drug	Yes	Diazepam-treated mice showed lower T_sc_ at 0 s (*p* = 0.001) and T_body_ from 0–270 s (*p* < 0.001–0.05), spend more time in open arms and had higher number of open arms entries. Diazepam-treated mice showed no differences between animals tested first and second, while having lower T_sc_ and T_body_, despite being more active. Anxiolytic effects were consistent across sexes, strains and handling techniques.	
(added) Animals tested as second and third will show higher impact of SIH than animal tested first in the cage.	Yes	DH	Animals tested second showed higher T_sc_ (*p* < 0.001) T_body_ (*p* < 0.001) and lower T_tail_ (*p* < 0.001), as well as animals tested third (T_sc_ (*p* < 0.001), T_body_ (*p* < 0.001) and T_tail_ (*p* < 0.001))
		VIT	Animals tested second showed higher T_sc_ (*p* < 0.001) T_body_ (*p* < 0.01) and T_tail_ (*p* < 0.001).
		EPM	Animals tested second had higher T_sc_ (*p* < 0.001) at 0 s and higher T_body_ (*p* < 0.01–0.05) from 0–60 s.

**Figure A1:**
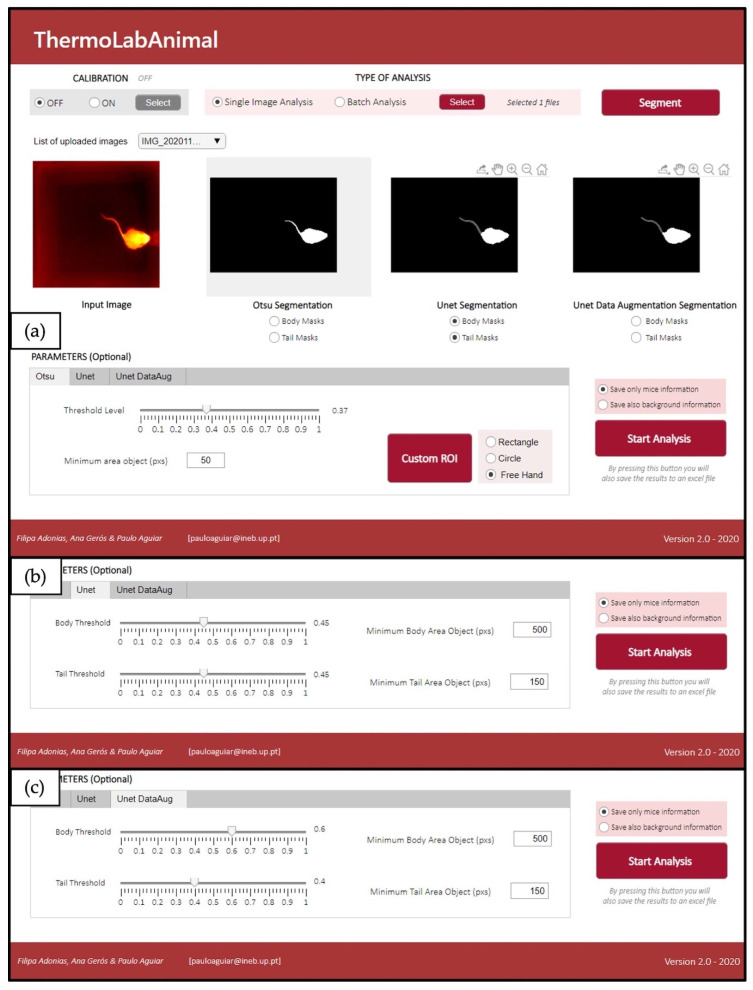
Image presenting ThermoLabAnimal 2.0 main interface, which allows automatic segmentation of the animal (body and tail) in two steps. First, it is possible to choose the type of analysis: single-image analysis or batch analysis. Second, parameters for each one of the segmentation methods can be fine-tuned: (**a**) in Otsu’s method, it is possible to adjust for threshold level (minimum area of pixels detected) and to manually select the region of interest (Custom ROI); (**b**) in the U-Net- based method it is possible to adjust body and tail threshold level (minimum area of pixels detected) to improve the quality of segmentation; (**c**) uses the same U-Net as in (**b**) but trained with an augmented dataset (which, in some cases, may provide improved results).

**Figure A2:**
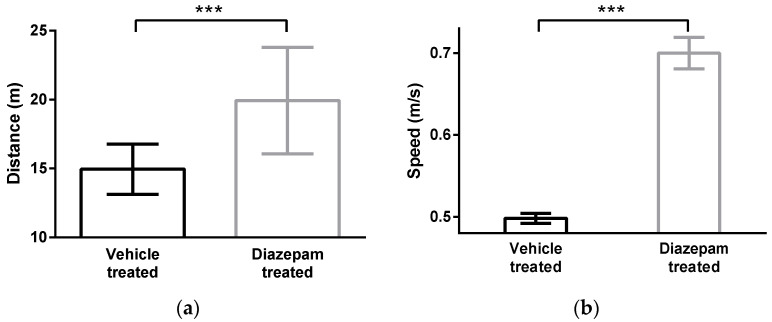
(**a**) Distance travelled and (**b**) average speed of traveling during 5 min EPM test, presented for C57BL/6J mice, separated for drug treatment (diazepam- and vehicle-treated mice). Error bars represent standard deviation, *** *p* < 0.001.

## Data Availability

The data presented in this study are openly available on FigShare (DOI:10.6084/m9.figshare.7771091).

## Appendix B

### Appendix B.1. Analysis and Statistics

To additionally analyze whether thermal responses in the EPM are result of motor activity or anxiety, we used ANY-maze version 6.34, to measure distance travelled and speed of the mice exploring Elevated Plus Maze apparatus. Due to technical difficulties with the videos (weak contrast between white BALB/cByJ mice and the background), we were only able to analyze the videos of black C57BL/6J mice.

All statistical tests were performed using SPSS (version 27.0). An independent *t*-test was used with drug treatment as independent variable.

### Appendix B.2. Results

We observed a statistically significant difference in the distance travelled between the two drug groups (t_(29)_ = 4.62, *p* < 0.001, Figure A2a), with diazepam-treated mice showing higher distance travelled ($\bar{x}$ = 19.9 m ± 3.9 m) as compared to vehicle-treated mice ($\bar{x}$ = 15.0 m ± 1.8 m). The same was observed for the speed of travel (t_(17)_ = 3.90, *p* = 0.001, Figure A2b), with diazepam-treated mice traveling faster ($\bar{x}$ = 0.7 m/s ± 0.019 m/s) as compared to vehicle-treated mice ($\bar{x}$ = 0.5 m/s ± 0.006 m/s).
